# The role of different nutrients in the prevention and treatment of cardiovascular diseases

**DOI:** 10.3389/fimmu.2024.1393378

**Published:** 2024-05-10

**Authors:** Zhi Tu, Jinfu Yang, Chengming Fan

**Affiliations:** Department of Cardiovascular Surgery, the Second Xiangya Hospital, Central South University, Changsha, China

**Keywords:** cardiovascular disease, nutrient, diet, antioxidant, omega-3 fat acid, fibre, antioxidants

## Abstract

Cardiovascular health is a hot topic around the world, and as the incidence of cardiovascular disease increases each year, people are increasingly focusing on the management of their heart health. Dietary and lifestyle changes as non-pharmacological treatments have been increasingly recognized as important in the prevention of cardiovascular disease and in reducing the risk of cardiovascular accidents. Awareness of different nutrients and their effects on cardiovascular health is important for establishing a good dietary pattern. This review summarizes the effects of the five major nutrients in the daily diet, namely carbohydrates, proteins, dietary fats, vitamins, and minerals, on cardiovascular health, and aims to provide a more comprehensive understanding of the effects of a healthy dietary pattern on cardiovascular health.

## Introduction

As the global incidence of heart disease continues to increase year on year, more people are becoming aware of their heart health management (1). Dietary and lifestyle changes, in addition to pharmacological interventions for those who already have the disease or are at risk of developing it, are increasingly recognized as a means of preventing and delaying the onset of the disease (2). The Mediterranean diet, a dietary pattern closely related to cardiovascular health, has been shown to reduce the burden of cardiovascular disease, cancer, diabetes, and obesity, as well as play a preventive role (3). The Mediterranean diet includes fish, monounsaturated fats in olive oil, fruits, vegetables, whole grains, legumes/nuts, and moderate alcohol consumption. Related studies have reported that this dietary pattern can also improve surrogate indicators of cardiovascular disease, such as waist-to-hip ratio, blood lipids, and inflammatory markers, and can even have the same effect as cardiovascular prevention and treatment drugs (4). Although the Mediterranean diet pattern it is currently unclear whether the Mediterranean diet confers cardiovascular disease benefits from its individual components or overall components, some studies have shown that increasing the intake of foods such as fruits, vegetables and dietary fibre and reducing the intake of saturated fatty acids, red meat and sugar can effectively reduce the risk of cardiovascular disease (5). At the same time, several studies have shown that the biologically active parts of many dietary components can be directly or indirectly involved in regulating biological metabolic and influencing the expression of hormones, receptors and other proteins (6). Diet is the main source of nutrient intake, and different dietary structures contain different nutrients, which can lead to individual differences in cardiovascular health (7). A low-fat diet reduces the risk of death and non-fatal myocardial infarction in people at increased cardiovascular risk, as demonstrated in a systematic review (8). Apart from this, dietary fibre (9) and antioxidants (10) are also closely related to the development of cardiovascular disease. These evidences suggest that, in addition to the study of dietary patterns, the role of different food nutrients in the treatment and prevention of cardiovascular diseases is a unique and valuable direction, and the study of the role and influence of different nutrients in the occurrence and development of cardiovascular diseases is of great significance for the prevention and treatment of cardiovascular diseases. In this review, we highlight the role of nutrients in the development, prevention, and treatment of cardiovascular diseases and discuss the epidemiological evidence on the relationship between nutrients and different types of cardiovascular diseases.

## The main nutrients found in the human body and how they relate to cardiovascular diseases

We know that the six essential nutrients in the human body are carbohydrates, proteins, fats, water, inorganic salts, and vitamins, and these nutrients are categorized into macronutrients and micronutrients based on their content (11). Carbohydrates, proteins and fats are the three main nutrients involved in supplying the body with energy and regulating metabolism. Carbohydrates serve as an important component that helps keep the body functioning and active (12); proteins, as nitrogenous organic compounds, are closely linked to growth, development and metabolism (13); dietary fat also serves as an essential nutrient in the growth and development of the body and is closely associated with a number of diseases (14). Similarly, vitamins and minerals are present in most foods as trace elements, and although they are small in proportion, they still play an important regulatory role in the organism (15). According to a scientific statement from the American Heart Association, dietary patterns are closely related to cardiovascular health (16). Many studies have shown that excessive weight gain during pregnancy increases the incidence of non-genetic obesity in the offspring (17). Meanwhile, according to a global review of food-based dietary guidelines (18), as we can see in the recognized healthy diets, their dietary structure has similar characteristics, such as the consumption of fibre-rich foods, the consumption of more fruit and vegetables, the control of processed foods and the reduction of salt intake. This highlights the importance of diet in improving cardiovascular health. We will review the nutrient components behind these dietary guidelines for healthy eating and their preventive and therapeutic effects on cardiovascular disease, and briefly describe some of the mechanisms that may be involved.

### Carbohydrates

**Figure 1:**
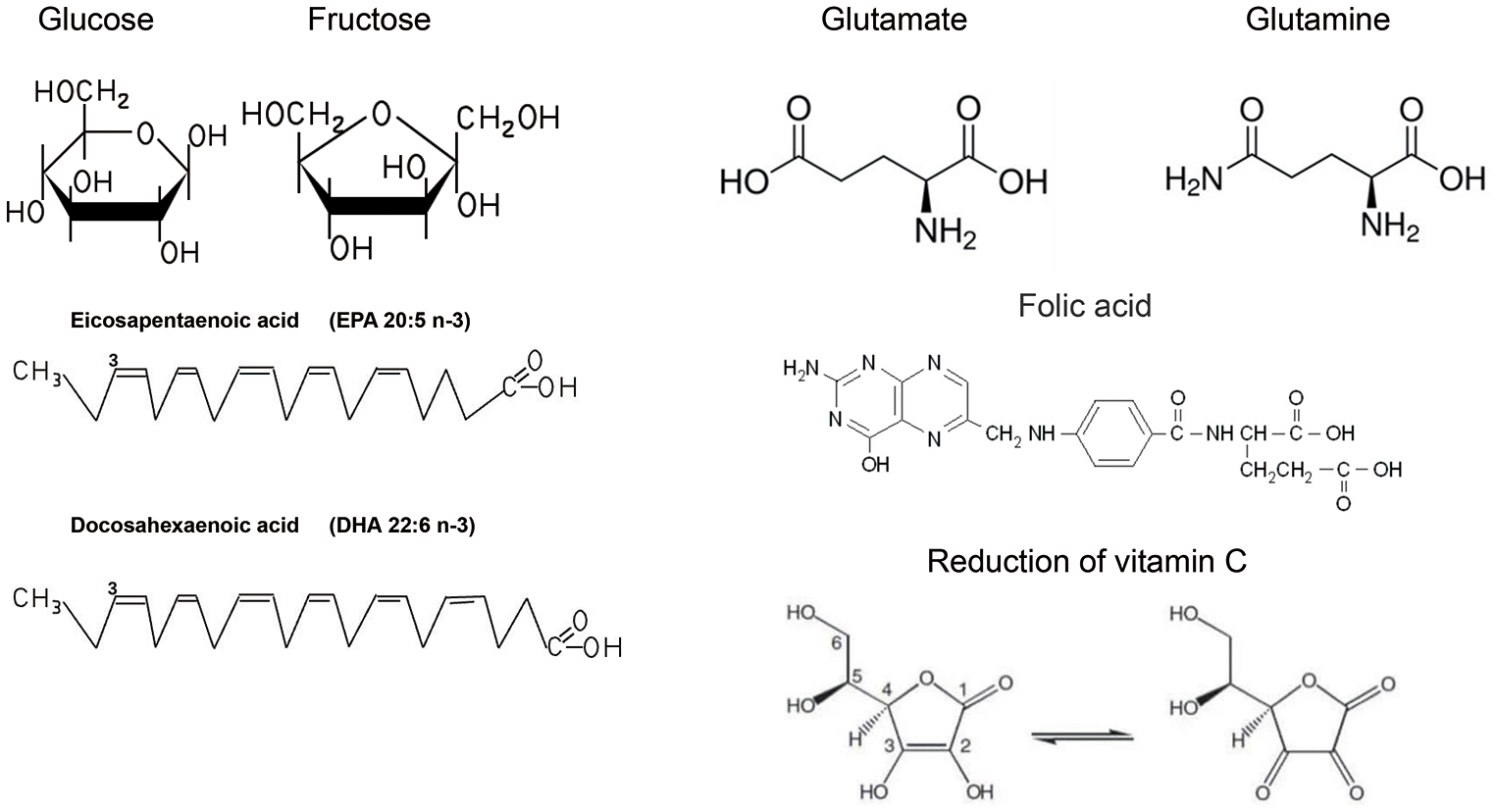
Common chemical formulae for nutrients.

In general, 50% to 70% of the energy required for human life is provided by the oxidative breakdown of sugars, and carbohydrates, as the main component of the general dietary structure (19), take on the role of providing sugars. Sugars in our daily diet include monosaccharides, disaccharides and complex sugars consisting of starch glycogen fibres (12). Starch, for example, is found in large quantities in staple foods such as cereals and wheat, and is broken down into glucose (**Figure 1**) by digestive enzymes in the digestive tract and absorbed as monosaccharides in the small intestine after the body has ingested such foods (20). The absorption of glucose in the small intestine is an energy-consuming process accompanied by the transport of sodium ions called sodium-driven sugar cotransporters (SGLTs) (21). There is another class of glucose transport proteins called concentration gradient dependent glucose transporters (GLUTs) (22). They are now known to be 12 in number and function in different parts of the body and are closely related to the development of disease (23).

Unbalanced dietary intake as well as some diseases can upset the metabolic balance in the body and may lead to metabolic syndrome (24). The metabolic syndrome, also known as the X-syndrome, was first used to describe the relationship between abdominal obesity and metabolic abnormalities, and subsequently it was proposed that the metabolic effects of aging, obesity, and sex hormone-related effects are associated with the risk of cardio genesis (25). A meta-analysis also confirmed that metabolic syndrome increases the risk of cardiovascular-related adverse events (26). Key features of the metabolic syndrome include dyslipidaemia, insulin resistance, hypertension, abdominal obesity and elevated levels of various inflammatory markers (27). Different types of carbohydrates have different effects on cardiovascular disease risk, and one study suggests that increasing intake of dietary fibre, as well as grains, in place of highly refined carbohydrates may have cardiovascular protective effects (28). Dietary fibre is a complex group of carbohydrates that are generally not digested and absorbed by the body due to the lack of enzymes in the body to digest fibre (29). But in fact, dietary fibre consists of both soluble and insoluble fibre fractions, which are intact in plants, whereas soluble dietary fibre is absorbed in the form of short-chain fatty acids out of the large intestine and the insoluble fraction cannot be digested (9). Whether soluble or insoluble, fibre plays an important role in cardiovascular health (30). The main roles that dietary fibre may play include bile acid salt metabolism, enzyme-substrate interactions, nutrient encapsulation, interactions with the mucus layer, and microbial degradation (31). The effect of these factors may improve blood glucose, lipid and insulin levels by influencing increased intestinal viscosity, faecal distension, etc., which reduces energy intake and promotes satiety, ultimately influencing cardiovascular disease occurrence (32). In addition, a class of functional dietary fibres, such as prebiotics, can lead to specific changes in the composition and/or activity of the gastrointestinal microbiota, which can benefit the health and wellbeing of the host (33). In addition to the effects of dietary fibre on cardiovascular health, many studies have focused on the relationship between carbohydrates on blood glucose, diabetes and obesity and have proposed a carbohydrate-insulin model (34). According to the Carbohydrate-Insulin Model (CIM) of obesity, a high-carbohydrate diet including refined grains and sugar intake produces a similar postprandial hyperinsulinaemia. Elevated insulin reduces all major metabolic fuels such as glucose, stimulates tissue uptake of glucose, inhibits the release of fatty acids from adipose tissue, inhibits hepatic production of ketone bodies, and promotes the deposition of fats and glycogen, thereby decreasing insulin levels and circulating fatty acid concentrations. This will result in promoting calorie deposition in fat cells rather than oxidation of lean tissue, thus increasing hunger, slowing the metabolic rate, or both, thus predisposing to weight gain (35). A randomized controlled trial showed that low-carbohydrate and low-calorie diets similarly reduced weight, waist circumference, fasting glucose, insulin, postprandial glucose changes, monocyte chemotactic protein-1, free fatty acids and fibroblast growth factor 21 (36). In another randomized controlled trial of carbohydrate diets and glycaemic control in people with diabetes, moderate carbohydrate restriction modestly improved glycaemic control and reduced circulating and intrahepatic triglyceride levels over and above the effects of weight loss itself (37). Blood glucose levels determine much of the impact of carbohydrates on cardiovascular disease. Through the tricarboxylic acid cycle, we know that when we consume more energy than our body needs, the excess sugar is stored in the body by being converted to fat, which in turn leads to obesity, one of the risk factors for cardiovascular disease (38). Blood glucose levels also affect the regulation of insulin, which is closely related to blood pressure. It influences the regulation of blood pressure by increasing sodium and water reabsorption in the kidneys and by affecting the diastolic function of vascular smooth muscle as well as the sympathetic nervous system (39). For diabetic patients, the intake of carbohydrates will be more cautious, because cardiovascular disease is one of the major complications of diabetes mellitus. In addition to the cardiovascular disease caused by hyperglycaemia mentioned above, diabetic patients are also accompanied by abnormalities in lipid metabolism and oxidative stress and other factors, which will gradually damage the endothelium of blood vessels as well as the heart itself, increasing the burden on the heart (40). Overall carbohydrates affect the health of the body, although there is no direct evidence that improving the quality of carbohydrate intake or simply consuming a low-carbohydrate diet improves cardiovascular health, both in terms of the type and quantity of carbohydrates consumed, carbohydrates are not only involved in the metabolism of substances, such as the tricarboxylic acid cycle. At the same time, carbohydrates influence endocrine functions, and some unabsorbed carbohydrates, such as dietary fibre, are involved in the absorption of substances, which also indirectly influences substance metabolism.

### Fats

Lipid is a general term for fats and lipids. Fat is often referred to as triglyceride, which is formed through the esterification of the hydroxyl group of glycerol with three identical or different fatty acids and is an important energy supply and energy storage substance in our body (41). Lipids include sterols, phospholipids, glycolipids and so on (42). Phospholipid molecules have unique hydrophilic and hydrophobic structures which form the basis of biological membranes, and phosphatidylinositol is a precursor of second messengers involved in intracellular signalling (43). Cholesterol is the precursor of a variety of biologically functional steroids, which, like phospholipids, is a fundamental structural component of cell membranes, and serves as a raw material for the synthesis of steroid hormones as well as being involved in the synthesis of vitamin D in the body (44).

Fatty acids are important components of fats, cholesterol esters and phospholipids, and are also an important component of food. Like other nutrients, fatty acids are classified as essential or non-essential, and essential fatty acids, such as linoleic acid and linolenic acid, cannot be synthesized due to the lack of the enzyme that desaturates these fatty acids in the human body (45). Fatty acids can also be classified according to their molecular structure, i.e. whether there are double bonds on the carbon chain of the fatty acid and can be classified as saturated fatty acids, unsaturated fatty acids, and polyunsaturated fatty acids (11). Currently, dietary unsaturated fatty acids (TFA) have been the focus of the effects of dietary fats on cardiovascular disease, including the effects of trans fatty acids (TFA) and polyunsaturated fatty acids (PUFA) on cardiovascular health.

**Figure 2:**
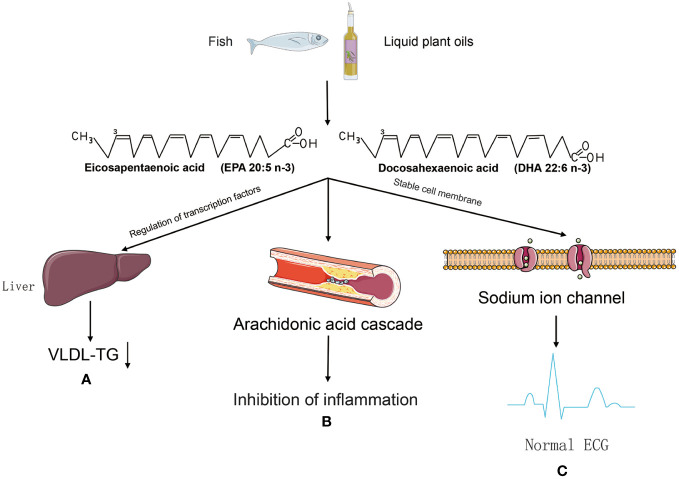
The mechanism of cardiovascular protection by PUFA. **(A)** PUFA may be involved in the regulation of transglutaminating factors to reduce VLDL-TG synthesis and improve atherosclerosis. **(B)** Metabolised by pro-inflammatory lipid mediators in the arachidonic acid (AA) cascade, but their active metabolites are thought to be weaker than the active metabolites of AA, thus tipping the balance in favour of inhibition of inflammation. **(C)** Omega-3 PUFA prevents arrhythmias caused by sustained sodium spillage in cardiomyocytes after myocardial infarction.

Regarding the mechanism of cardiovascular protection by PUFA (**Figure 2**), the main ideas include the regulation of blood lipid levels to improve atherosclerosis (46), participation in the production of anti-inflammatory substances (47), and the maintenance of biofilm stability (48). Hyperlipidaemia is an important risk factor for cardiovascular disease, and studies have shown that patients with hyperlipidaemia who take omega-3FAs, including DHA and EPA (**Figure 1**), have significantly lower triglyceride levels (49). And there is evidence that the use of prescription (EPA DHA or EPA only) doses of 4 g/d (>3 g/d total EPA and DHA) is an effective and safe option for lowering triglycerides, either as monotherapy or as adjunctive therapy to other lipid-lowering agents (49). The role of EPA and DHA in reducing hepatic VLDL-TG synthesis, as well as enhancing TG clearance, may be related to the reduction of VLDL-TG synthesis in the liver, which has been demonstrated in relevant studies (50). Although the molecular mechanisms underlying this significant reduction are not fully understood, potential mechanisms may be related to the regulation of transcription factors involved in hepatic fatty acid uptake, synthesis, and oxidation, as well as transcription factors involved in TG synthesis and VLDL assembly (51).

High dietary intake of omega-3 fatty acids leads to appropriate accumulation of these fatty acids in cell membranes and circulating lipids, and phagocytosis by cell membranes affects the fluidity and biophysical properties of lipid rafts (52), they act as modulators of protein function and are also involved in signalling pathways (53). The mechanisms of anti-inflammatory effects of EPA and DHA may include alteration of cell membrane phospholipid fatty acid composition, disruption of lipid rafts, inhibition of pro-inflammatory transcription factor NF-κB activation, resulting in reduced expression of inflammatory genes and activation of the anti-inflammatory transcription factor peroxisome proliferator-activated receptor γ (54). Specifically, cell membranes enriched with omega-3 PUFA disrupted recruitment and initiation of toll-like receptor-4, which triggers anti-inflammatory effects through downregulation of the NF-κB pathway, and inhibition of the pro-inflammatory cytokines in aortas (IL6, TNFα, IL1, and c-cytokine). IL6, TNF-α, IL1, and c-reactive protein (CRP)) in the aorta, reducing the infiltration of inflammatory cells (macrophages) into the intima, thereby reducing atherosclerosis and plaque formation (55). At the same time omega-3 fatty acid, may have an effect on platelet aggregation as well as anticoagulant function (56). Thromboxane A2 (TXA_2_) and prostaglandin E2 (PGE_2_) produced by platelets in physiological haemostasis promote platelet aggregation and contraction, thereby promoting coagulation as well as thrombosis (57). Prostacyclin 2 (PGI_2_), which is produced by the endothelial cells of blood vessels, inhibits the formation of blood clots (58). Whereas prostacyclin 2 production by vascular endothelial cells inhibits thrombosis, EPA appears to reduce the ability of endothelial cells to produce PGI2 in two ways: by lowering arachidonic acid levels in the cellular pool of phospholipid precursors, and by acting as an inhibitor of prostaglandin production (59), which is thought to be important evidence that consumption of EPA-enriched seawater fish can reduce the risk of myocardial infarction (60). A cohort study analysed a dose-response relationship between higher TFA intake and increased risk of cardiovascular disease (61). Another meta-analysis showed that higher concentrations of marine-derived omega-3 PUFA biomarkers were associated with significant reductions in cardiovascular disease, coronary heart disease, and total mortality (62). Several studies appear to support this claim, (63) showed that the effect of TFA on cardiovascular disease may affect the cardiovascular system by lowering HDL. Another study found that introducing or replacing saturated and trans fats with unsaturated fatty acids in obese or overweight non-diabetic older adults was beneficial in reducing cardiovascular risk (64). Omega-3 fatty acids including docosahexaenoic acid (DHA) and eicosapentaenoic acid (EPA) have been shown to significantly reduce the risk of sudden cardiovascular death and all-cause mortality, as well as being used to treat hyperlipidaemia and hypertension (65). The importance of essential fatty acids (EFAs) has been recognized in the fields of cardiac science and cardiology, and polyunsaturated fatty acids are considered important multifunctional mediators for improving and maintaining human health throughout the life cycle (66). In general, dietary fatty acids have an impact on the development and progression of cardiovascular disease in a number of ways. Fat intake influences lipid metabolism. Excessive fat intake is also associated with a higher incidence of obesity. Some fatty acids, such as saturated and trans fatty acids, may be associated with atherosclerosis through oxidative stress and metabolic effects. Fatty acids can also affect the stability of cell membranes and some of the ion channels in the membranes, which can lead to electrophysiological abnormalities in cardiomyocytes and ultimately to cardiac arrhythmias. On the other hand, some of the anti-inflammatory, anti-thrombotic and membrane stability-enhancing properties of unsaturated fatty acids may play a role in the prevention of cardiovascular disease.

### Proteins

Proteins are composed of amino acids as the basic unit, the process of absorbing proteins in food needs to hydrolyse proteins into oligopeptides and amino acids under the action of digestive enzymes in order to be absorbed by the human body, and this process of proteins will lose the original structure and function, so we can think that the actual value of proteins is determined by the type and amount of amino acids they contain (67). Amino acids are classified as essential or non-essential according to whether they can be synthesized by the body itself (68). Essential amino acids include leucine, isoleucine, threonine, valine, lysine, methionine, phenylalanine, tryptophan, and histidine (69). In addition, although arginine can be synthesized by the human body, studies have reported that long-term insufficient intake can cause negative nitrogen balance, so it is also classified as an essential amino acid (70). Amino acids are organic compounds that are essential for sustaining life, providing raw materials for biosynthesis and energy for living activities. In addition to being raw materials for biomass and sources of energy, amino acids are involved in key pathways for cell growth, metabolism and immunity (71). An important mechanism for regulating protein synthesis is the mammalian target of rapamycin (mTOR) signalling pathway. The mTOR system includes the rapamycin-sensitive complex 1 (mTORC1), which is activated by glutamine (Gln)(**Figure 1**), arginine (Arg) and leucine (Leu) and activates protein synthesis by phosphorylating eIF4E-binding protein 1 (4E-BP1) and ribosomal protein S6 kinase 1 (S6K1) to activate protein synthesis (72). Arginine is involved in a wide range of biological processes. The synthesis of urea and creatine uses arginine to balance nitrogen and meet the metabolic needs of muscle. As a free amino acid, arginine is a precursor for the formation of nitric oxides(NO) and Gln, important neurotransmitters that regulate vascular tension and nerve conduction (73). The process of methylation of arginine is catalysed by protein arginine methyltransferases (PRMTs).Increased PRMT activity leads to enhancement of the metabolite asymmetric dimethylarginine (ADMA), both of which are associated with an increased risk of cardiovascular disease because they tend to inhibit nitric oxide synthase (NOS) (74). Accumulation of the endogenous NOS inhibitor ADMA (significantly reduces the synthesis of the potent vasodilator NO and contributes to the development of cardiovascular disease. Glutamine is structurally similar to glutamic acid, with the carboxylic acid group of the side chain replaced by an amide, and is an amino acid used in protein synthesis (75). Glutamine is a dietary non-essential amino acid and is the most abundant amino acid involved in the synthesis of all non-essential amino acids and proteins. Muscle tissue produces the most glutamine in the body, accounting for approximately 90% of all glutamine synthesised. Although the liver also synthesises glutamine, its main function is to regulate glutamine levels. The brain and lungs also produce small amounts of glutamine (76). Gln is a key amino acid in the regulation of glucose stability and insulin sensitivity, and Gln levels affect the inflammatory response in skeletal muscle and regulate the expression of the adaptor protein GRB10, an inhibitor of insulin signalling. In addition, systemic elevation of Gln improves insulin sensitivity and restores glucose homeostasis in a mouse model of obesity (77). Gln levels influence the inflammatory response in skeletal muscle and modulate the expression of the adaptive protein GRB10, which inhibits insulin signalling. Furthermore, systemic elevation of Gln improves insulin sensitivity and restoration of glucose homeostasis (77). The anthracycline antibiotic doxorubicin (DOX) is a cardiotoxic antineoplastic drug that can cause free radical and oxidative stress. Gln supplementation significantly reduces cardiac lipid peroxide levels and increases peroxidase and glutathione levels, thereby protecting cardiac function (78).

The amount of protein in foods varies greatly and is commonly found in animal sources such as meat, dairy products, and eggs. Some plants also contain significant amounts of protein such as legumes. The effects of protein on human health, especially on cardiovascular health, have been controversial, and there is no definitive conclusion on the merits of animal and plant sources of protein (79). The benefits of plant-based sources of protein began to spread among vegetarians, and prospective studies have shown that consumption of a vegetarian diet is associated with a reduced risk of coronary artery disease (80) and other cardiovascular disease (81). However, plant-based proteins also have limitations. For example, most plant-based proteins are deficient in some essential amino acids, such as lysine in cereals and tryptophan in legumes (82). Vegan diets may also be deficient in certain nutrients such as vitamin B12, iron and zinc (83). The role of animal proteins is also twofold. On the one hand, animal-derived amino acids provide a sufficiently complete range of essential amino acids, which are thought to promote muscle growth and development (84). On the other hand, the intake of proteins of animal origin, especially red meat, and processed meat products, can lead to an increased risk of cardiovascular disease (85). A cohort study demonstrated that higher intake of processed meat, unprocessed red meat or poultry was significantly associated with a small increase in the risk of developing cardiovascular disease (86). More evidence is needed on the advantages and disadvantages of plant and animal proteins and their relationship to cardiovascular disease, and the selection of proteins in the daily diet needs to be based on a combination of factors and individualized dietary regimens.

### Micronutrients

Micronutrients include vitamins and minerals, which are present in the human body at very low levels compared to the macronutrients mentioned above (87). Most micronutrients cannot be synthesized by the human body, and the body obtains them mainly through the daily diet or nutritional supplements, etc. There are also micronutrients that can be synthesized and utilized by human beings under certain circumstances. In the case of vitamin D, for example, 7-dehydrocholesterol in the skin under solar ultraviolet conditions is eventually converted to vitamin D3 and metabolized in the liver to 25-hydroxyvitamin D3, which is ultimately present and biologically active in the kidneys as 1,25-dihydroxyvitamin D3 (88). Although nutrients such as vitamin D can be synthesized and utilized on their own, because they require the involvement of ultraviolet light, factors such as the season, the amount of time spent in the sun during the day, age, and the use of sunscreens can affect the skin’s production of vitamin D3 (89). Therefore, although some of the nutrients can be utilized synthetically on their own, it is still necessary to obtain adequate nutrients through food, subject to several factors (90).

Vitamins are a diverse group of micronutrients that are broadly classified into lipid-soluble (91) and water-soluble vitamins (92) based on their physicochemical properties. Fat-soluble vitamins include vitamins A, D, E, and K. These vitamins are soluble in lipids and organic solvents and are absorbed and stored in the body along with lipids, usually in the liver (93). It has been suggested that impaired lipid absorption may cause deficiencies in these vitamins (94). Water-soluble vitamins include the B vitamins (e.g. B1, B2, folic acid, etc.) (95) and vitamin C (96). These vitamins are more dependent on dietary sources than fat-soluble vitamins due to their physio-chemical properties and the fact that they rarely accumulate in the body, and in case of excess they are excreted in the urine (97). The specific classification of vitamins and their corresponding physiological functions and sources of acquisition have been described in detail in other studies (11), and in this paper, we will focus on some of these vitamins, which are common and supported by reliable evidence, and analyse their role in the treatment and prevention of cardiovascular disease.

**Figure 3:**
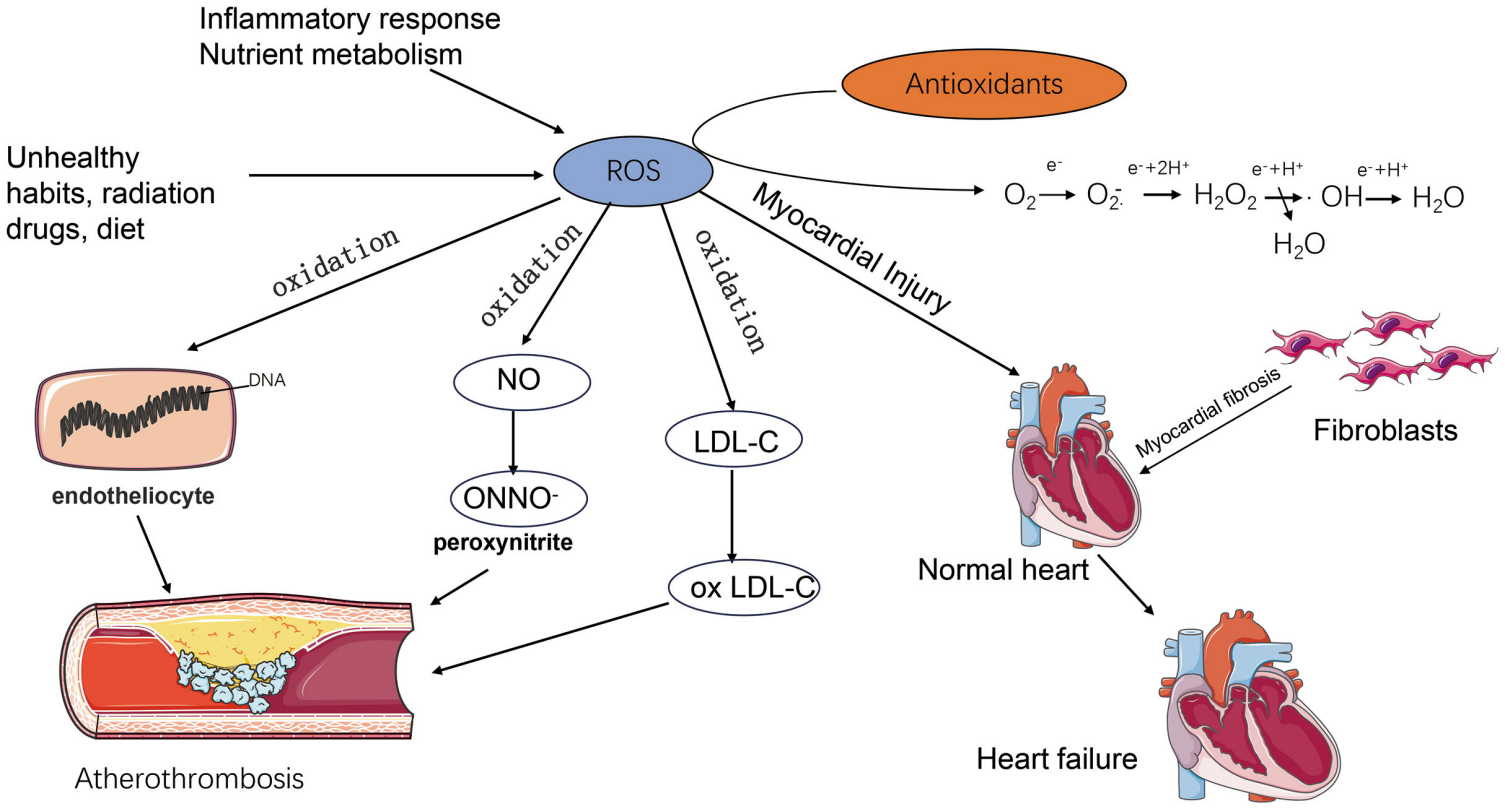
The increase of ROS caused by oxidative stress directly leads to the oxidation of proteins and nucleic acids, which directly destroys the cellular structure and leads to the direct damage of endothelial cells. ROS can oxidize nitric oxide and produce peroxynitrite, which further damages the normal cellular structure and function. Oxidative stress oxidises LDL (ox LDL), which can more easily penetrate the endothelium of the arteries and induce the formation of lipid plaques, which can lead to inflammatory reactions in the vessel wall, and thus increase the risk of cardiovascular events. inflammatory response, thus increasing the risk of cardiovascular events. Oxidative stress can lead to apoptosis and necrosis of cardiomyocytes through direct damage to cardiomyocytes, and promote fibroblast proliferation, leading to diastolic and systolic dysfunction as well as myocardial fibrosis, which ultimately leads to the development of heart failure.

Vitamin C (**Figure 1**) is a familiar antioxidant with reducing properties that protect cells from oxidative damage by counteracting oxidative stress and help improve cardiovascular health. In order to understand the mechanisms of oxidative stress on cellular damage, we need to recognize the process of biological oxidation (98). We know that the process of oxidative breakdown of chemicals in living organisms is called biological oxidation, most typically in the mitochondrial oxidation system, where the main products are water and carbon dioxide, a process that requires the consumption of oxygen and the production of ATP as well as heat (99). The purpose of biological oxidation is the complete oxidation of nutrients such as sugars, fats, and proteins in food, which is a process in which hydrogen is removed and eventually transferred to oxygen molecules to form water molecules, which will be briefly outlined (100). Firstly, biological oxidation requires a variety of hydrogen and electron transferring components, including nicotinamide adenine dinucleotides, flavin nucleotide derivatives, as well as ubiquinone, also known as coenzyme Q, and cytochromes (101). The transfer of electrons and hydrogen from the above substances is also dependent on the oxidative respiratory chain consisting of a complex of proteins with the ability to transfer electrons, NADH, FADH2, which passes through a series of oxidoreductase systems in the oxidative respiratory chain. Firstly, oxidative respiration requires a variety of hydrogen- and electron-transferring components, including nicotinamide adenine dinucleotide, flavin nucleotide derivatives, as well as ubiquinone and cytochromes. The transfer of electrons and hydrogen from the above-mentioned substances is also dependent on the oxidative respiration chain consisting of a complex of proteins with the ability to transfer electrons, NADH, FADH2, which passes through a series of oxidoreductase systems in the oxidative respiration chain. Firstly - the release of electrons from NADH, FADH2 on the stromal side of the mitochondrial membrane, the electrons pass through the oxidative respiratory chain (this process includes complexes I-IV), these complexes receive and release energy through the function of the proton pump of the complexes to transport H+ from the mitochondrial matrix to the cytoplasmic side, as protons are not free to pass through the inner membrane of the mitochondria to return to the stroma, which in turn creates an electrochemical gradient and drives the complexes V, i.e. ATP synthase, to synthesize ATP. at the same time the oxidative respiratory chain ultimately transfers electrons to oxygen and reduces oxygen to water at complex IV (101). In the process of biological oxidation, the mitochondria exist but the electron transfer process, when a single electron is directly transferred to the oxygen will generate reactive oxygen species rather than simply through the oxidative respiratory chain to oxygen to generate water, so that a series of products such as superoxide anion, hydrogen peroxide, hydroxyl radicals collectively known as the reaction reactive oxygen species (102). For reactive oxygen species (ROS), a large number of studies have demonstrated their effects on cellular function (103), and although there are reports showing that small amounts of ROS, and under specific circumstances, promote cell proliferation (104), there is more evidence that accumulation of large amounts of ROS impairs cellular function and leads to cell death (105). In most studies we have found that the cardiovascular effects of reactive oxygen species (ROS) focus on damage to the vascular endothelium (106). On the one hand, the increase in ROS due to oxidative stress can lead directly to the oxidation of proteins and nucleic acids, which can directly damage the cellular structure and lead to direct damage to the endothelium (107). On the other hand, ROS can oxidize nitric oxide, thereby reducing its bioavailability, nitric oxide is an important vasodilatory factor (108), while peroxynitrite a product of nitric oxide oxidation, also belongs to the category of ROS and further damages the normal structure and function of cells (109). In addition, oxidative stress can cause apoptosis and necrosis of cardiomyocytes through direct damage to cardiomyocytes (110), and may also promote the proliferation of fibroblasts leading to diastolic and contractile dysfunction of the myocardium and fibrosis of the myocardium ultimately leading to the development of heart failure (111). Oxidative stress is also strongly implicated in atherosclerosis. Oxidative stress increases the risk of cardiovascular events by oxidising LDL so that it can more readily cross the arterial endothelium and induces the formation of lipid plaques leading to an inflammatory response in the vessel wall (112). Oxidative stress can also activate platelet formation, increasing the risk of thrombosis (113). Although there are antioxidant systems in the body such as superoxide dismutase (SOD) (114), glutathione peroxidase (GPx) (115), etc., which are capable of scavenging oxygen radicals and other oxidative substances to maintain the redox balance in the body, but subject to the individual’s level of health, living habits, living environment and other influences, the body itself oxidative stress generated by free radicals may exceed the scavenging capacity of antioxidant enzymes (116), which at this point requires additional supplementation of antioxidants in order to meet the body’s needs (117) (**Figure 3**). Vitamins are used in many dietary guidelines and health recommendations as excellent antioxidants, and the convenience of their role as antioxidants against oxidative stress may be related to their own structure as well as their reducing properties (118). Firstly, vitamins as antioxidants can neutralize the activity of free radicals by directly donating electrons to them, e.g. vitamin C (Ascorbic acid) (119), and in addition, e.g. vitamin E, which reacts with oxidized lipids and prevents the lipid peroxidation chain reaction, protects cell membranes from damage (120). In addition, vitamin C regenerates oxidized vitamin E and vitamins modulate the activity of antioxidant enzymes indirectly affecting the antioxidant capacity of cells (121). In general, vitamins, as a class of antioxidants, help cells to remove reactive oxygen species generated by oxidative stress by donating electrons to eliminate oxidative regenerative oxidants and by increasing the activity of the body’s own antioxidant enzyme system, which maintains the redox balance of the cells and protects the cells from the damage caused by oxidative stress. It is worth mentioning that although many clinical studies have shown that the consumption of vitamin-rich foods such as vegetables and fruits can reduce cardiovascular disease incidence and mortality, however there is only limited evidence to support the benefits of vitamin and mineral supplementation in the prevention of cancer or cardiovascular disease (122).

Minerals are also an important class of micronutrients that play an important role in metabolism, growth and development (123). The 10 essential metal elements include sodium (Na), potassium (K), magnesium (Mg), calcium (Ca), manganese (Mn), iron (Fe), cobalt (Co), copper (Cu), zinc (Zn), and molybdenum (Mo) (124). Sodium and potassium are important substances involved in the metabolism of substances in the body. In addition, they are important components in maintaining homeostasis in the body’s internal environment (125). Sodium and potassium are distributed differently in the body. Sodium is mainly distributed in the extracellular fluid, whereas potassium is distributed more in the intracellular fluid, and the intracellular potassium concentration is as high as 140-150 mmol/L, which accounts for 98% of the total amount of potassium in the body (126). The main reason for the different ionic compositions in the cell is the presence of a sodium pump (known also as Sodium–Potassium-ATP-ase) in the cell membrane, in which hydrolysis of one molecule of ATP is accompanied by pumping three sodium ions out of the cell and two potassium ions into the cell (127). Sodium and potassium metabolism is regulated by blood pressure, the antidiuretic hormone aldosterone system and activation of β2-adrenergic receptors (125). Sodium, as an essential nutrient in our daily diet, is not only an inorganic salt necessary for the maintenance of life activities, but it is also closely related to the occurrence and prevention of cardiovascular disease (128). We consume sodium ions through salt in our daily diet, and related studies have reported the mode of sodium ions in the regulation of blood pressure and concluded that imbalance of sodium balance is the main cause of hypertension, and in some salt-sensitive people, plasma sodium levels are even more strongly correlated with blood pressure (129). Potassium ions may influence the regulation of blood pressure in relation to the homeostasis of sodium ions in the body (130). Calcium and magnesium are often compared together due to their physicochemical characteristics. Mg^2+^ is not only a cofactor for a number of enzymes in the body it also acts as a regulator of potassium and calcium ion channels and antagonises the function of calcium (131). Calcium ions are involved in the regulation of a variety of ion channels, such as small conductance Ca^2+^-activated K (SK) channels These channels play a key role in the regulation of cardiac excitability, and SK channels play a key role not only in the healthy heart but also in diseases such as atrial fibrillation (AF), ventricular arrhythmias and heart failure (HF) (132). In addition to its role in influencing blood pressure, related animal experiments suggest that modulation of Ca processing may be one of the molecular mechanisms underlying the effects of salt intake on myocardial function in hypertensive patients (133). It has also been shown that a high salt diet is associated with an increase in salt-induced expression of kinase 1, which may be associated with the activation of genes involved in left ventricular hypertrophy leading to alterations in myocardial mechanical properties (134). Reports have also shown that potassium, magnesium, and calcium intake also have an effect on blood pressure (135). Magnesium ions may be involved in the regulation of the vasodilator prostaglandin E1, and reduced synthesis of this substance under low magnesium conditions may induce cardiovascular disease (136). These ions may also affect the diastolic function of the myocardium which can lead to heart failure (137). Clinical results may contradict current theories, such as one study that showed insufficient evidence to support the use of potassium, magnesium, and calcium ions to effectively lower blood pressure (138). A prospective cohort study of 28,886 U.S. women showed a potential role for calcium intake in the primary prevention of hypertension and cardiovascular complications, but there was no direct evidence of changes in blood pressure with calcium supplementation (139). A prospective cohort study of 28,886 U.S. women showed a potential role for calcium intake in the primary prevention of hypertension and cardiovascular complications, but there was no direct evidence of changes in blood pressure with calcium supplementation (139).

In addition to the metal ions mentioned above, among the metal ions essential to the human body, the elements iron and zinc play an essential role in the human body, even though the elements in front of their content in the human body are small. Iron in food is mainly absorbed through heme iron in animal meat and non-heme iron in some plants and grains (140). Iron absorption occurs mainly in the duodenum and the upper part of the jejunum. Iron is absorbed by the body mainly in the form of divalent iron ions. Vitamin C can reduce trivalent iron to divalent iron due to its strong reducing properties, thus promoting iron absorption (141). Iron is a key metal element in the synthesis of haemoglobin. When there is an imbalance in iron intake, circulation, storage and loss, iron deficiency anaemia results, which is associated with a decrease in haemoglobin, myoglobin and cytochrome levels (142). Iron imbalances in the body include iron deficiency and iron overload and are present in a variety of cardiovascular diseases such as heart failure and myocardial infarction (143). Zinc is a component of many enzymes in the body and is involved in the metabolism of substances as well as other life activities (144). Also, zinc is found in a structure called zinc finger and binds to specific sequences of DNA or RNA to regulate biological processes such as gene expression, DNA replication and repair (145). Zinc deficiency increases the formation of ROS and causes significant oxidative damage to all important biological macromolecules such as DNA, proteins and membrane lipids, so the maintenance of a certain concentration of zinc in the body by an organism is closely related to heart health (146).

## Epidemiology of nutrients in CVD

We have described the role played by different nutrients in cardiovascular disease and analysed some of the possible mechanisms involved, and then we will further analyse their relationship from an epidemiological point of view (**Table 1**).

**Table 1:** Human epidemiological studies showing associations between nutrients and CVD.

Ref.	Nutritional conditions	Population	Results
Heart failure			
(147)	Breakfast cereal intake	Individuals from the Physicians' Health Study I(n=21376)	A higher intake of whole grain breakfast cereals is associated with a lower risk of HF
(148)	High-protein diet	Individuals from a randomized controlled study(n=76)	A high-protein diet may be more effective in reducing cardiometabolic risk in this population
(149)	Omega-3 PUFAs supplements	Individuals from double-blind, placebo controlled, cross-over trial (n=31)	Short term treatment with omega-3 PUFAs in subjects with stable ischemic HF improved inflammatory and fibrotic status as well as endothelial function in parallel with systolic and diastolic performance of left ventricle
(150)	4000 IU vitamin D daily	Individuals from an investigator-initiated, multicenter, prospective, randomized, placebo-controlled trial (n=73)	A daily vitamin D dose of 4000 IU for chronic HF appears to be safe and did improve the 6-min walk distance, symptoms, and left atrial diameter at 6 months.
Hypertension			
(151)	Very low carbohydrate (VLC)	Adults with a triple multimorbidity (hypertension, prediabetes, or type 2 diabetes, and overweight or obesity)	the VLC diet resulted in greater improvements in systolic blood pressure, glycemic control, and weight over a 4-month period compared with the DASH diet
(152)	Fiber and protein	An 8-week factorial study of parallel design (n=41)	Dietary protein and soluble fiber supplements lower blood pressure additively in hypertensives.
(153)	Sodium reduction	Of 412 participants, 57% were women, and 57% were black; mean age was 48 years, and mean SBP/diastolic BP was 135/86 mm Hg.	The combination of reduced sodium intake and the DASH diet lowered SBP throughout the range of pre- and stage 1 hypertension, with progressively greater reductions at higher levels of baseline SBP.
Coronary artery disease			
(154)	Fish intake	Postmenopausal women enrolled in the Estrogen Replacement and Atherosclerosis Trial (n = 229)	Consumption of fish is associated with a significantly reduced progression of coronary artery atherosclerosis in women with coronary artery disease.
(155)	Vegetable intake	Men who were free of heart disease, stroke, or cancer (n=15220)	A negative association between vegetable intake and risk of coronary heart disease.
(156)	300 000 IU of vitamin D was administered orally 12 hours before PCI	Patients undergoing elective PCI (n=99)	Mean difference in CK-MB between 8 hours and 24 hours was significantly lower in the vitamin D group. The mean difference in hs-CRP was significantly lower in the vitamin D group
(157)	Recommended daily allowance (RDA) of dietary calcium, zinc, and iron	Individuals from an matched case-control study which was carried out at Shahid Gangalal National Heart Center (n=466)	A significant inverse association of dietary zinc intake above RDA indicates the potential protective effect of higher dietary zinc against CAD. However, causal odds of CAD are inconsistent across the median or RDA of calcium and iron intakes.
Arrhythmia			
(158)	Antioxidants	–	Antioxidants might be useful as adjuvants in controlling reperfusion induced arrhythmias following therapeutic alteplase thrombolysis
(159)	Marine omega-3 fatty acids and vitamin D	An ancillary study of a 2 × 2 factorial randomized clinical trial involving 25 119 women and men aged 50 years or older without prior cardiovascular disease, cancer, or atrial fibrillation (AF)	Treatment with EPA-DHA or vitamin D3, compared with placebo, resulted in no significant difference in the risk of incident AF over a median follow-up of more than 5 years
Inherited cardiovascular conditions			
(160)	Multivitamin	Individuals from a population-based Atlanta Birth Defects Case-Control Study	Periconceptional multivitamin use is associated with a reduced risk for conotruncal defects

HF, Heart failure; PUFA, polyunsaturated fatty acid; DASH, Dietary Approaches to Stop Hypertension; SBP, Systolic Pressure; PCI, percutaneous coronary intervention; CAD, Coronary Artery Disease; EPA, eicosapentaenoic Acid; DHA, docosahexaenoic acid.

### Heart failure

Different nutrients all have an important impact on cardiovascular health. A study evaluated prospectively the association between breakfast cereal intake and incident HF among 21 376 participants of the Physicians’ Health Study I. The result demonstrate that a higher intake of whole grain breakfast cereals is associated with a lower risk of HF (147). In a randomized controlled study, results suggest that a high-protein (30% protein, 40% carbohydrates, and 30% fat) diet may be more effective in reducing cardiometabolic risk (148). In a double-blind, placebo-controlled crossover trial with 31 patients with ischaemic heart failure, the intervention group was given Omega-3 PUFAs (2 g daily for 8 weeks). The results showed improvements in the inflammatory and fibrotic state of the left ventricle, as well as endothelial function, and left ventricular systolic and diastolic function in the intervention group (149). Vitamin D intake at safe doses showed no improvement in endothelial cells in a randomised controlled trial, but improved 6-minute walking distance, symptoms and left atrial internal diameter at 6 months in patients with heart failure (150). The development of chronic heart failure is a long-lasting process, and although there has been growing evidence that a healthy diet can improve symptoms as well as cardiac function in patients with heart failure, more evidence is needed to confirm this.

### Hypertension

Low-carbohydrate diets show relevance in improving blood pressure in hypertensive patients. A randomized trial comparing a very low-carbohydrate (VLC) diet vs a Dietary Approaches to Stop Hypertension (DASH) diet. And the study showed the VLC diet resulted in greater improvements in systolic blood pressure, glycaemic control, and weight over a 4-month period compared with the DASH diet (151). In addition, dietary protein and soluble fibre supplementation was found to further reduce blood pressure in hypertensive patients in an 8-week, parallel-design, factorisation study (152). In a study of DASH-sodium (dietary patterns, sodium intake and blood pressure), the low-sodium-DASH diet was effective in reducing systolic blood pressure in prehypertension and stage 1 hypertension, and the reduction was greater at higher baseline systolic blood pressure levels (153).

### Coronary artery disease

A prospective cohort study of postmenopausal women (n = 229) participating in the Estrogen Replacement and Atherosclerosis trial showed higher fish consumption was also associated with smaller decreases in minimum coronary artery diameter and fewer new lesions (154). In addition some studies have reported vegan diet significantly reduces high-sensitivity C-reactive protein, an indicator of risk for adverse outcomes, by 32% compared to the American Heart Association diet (161). Although there was also no significant difference in glycaemic control measures between the two dietary groups. LDL cholesterol was 13% lower on the vegan diet compared with the American Heart Association diet. There were no significant differences in other lipid parameters. 15,220 men who were free of heart disease, stroke or cancer at baseline were investigated in a 12-year follow-up. The study showed men who consumed at least 2.5 servings/day of vegetables had a relative risk (RR) of 0.77 (95% CI: 0.60-0.98) for CHD (155). The results suggest an inverse association between vegetable intake and risk of CHD. In a randomized clinical trial investigating the role of vitamin D in preventing myocardial injury after selective percutaneous coronary intervention, we found that the mean difference in CK-MB between 8 hours and 24 hours was significantly lower in the vitamin D group. The mean difference in hs-CRP was significantly lower in the vitamin D group (156). For the study of dietary mineral intake for cardiovascular disease, a matched case-control study which was conducted to determine the causal effect of dietary intake of calcium, zinc and iron on coronary artery disease in Nepalese men showed a significant inverse association of dietary zinc intake above recommended daily allowance (RDA) indicates the potential protective effect of higher dietary zinc against CAD (157). Interestingly, the causal odds of CAD being caused by different median or RDA values for calcium and iron intake were not consistent. This may require cohort studies with larger sample sizes and randomised clinical trial studies.

### Arrhythmia

Arrhythmias involve excitability of cardiac machine cells, electrolyte abnormalities, and abnormalities in the cardiac conduction system. Although the number of related literature reports is smaller than that of other cardiovascular diseases, many studies have shown the correlation between diet and arrhythmia. A research showed the effect of antioxidant vitamins on biochemical changes and arrhythmias induced by reperfusion before and after therapeutic thrombolysis (Actilyse). Compared with patients taking placebo, patients taking antioxidants had significantly lower rates of premature ventricular contractions, atrial fibrillation, ventricular tachycardia, and first-degree atrioventricular block and increased plasma antioxidant properties. These findings suggest that antioxidants might be useful as adjuvants in controlling reperfusion induced arrhythmias following therapeutic alteplase thrombolysis (158). Another randomised clinical trial on the effects of supplementation with marine Omega-3 fatty acids and vitamin D on the incidence of atrial fibrillation showed no significant difference in the incidence of atrial fibrillation at a median follow-up of more than 5 years with EPA-DHA or vitamin D3 treatment compared to a placebo group in adults aged 50 years or older (159).

### Inherited cardiovascular conditions

Inherited cardiovascular diseases (CVDs) are a complex group of diseases that are caused by genetic mutations or hereditary factors. The effect of folic acid (**Figure 1**) on congenital heart disease has been the main subject of research on the relationship between cardiovascular disease and nutrients. A study in 1964 showed a higher rate of congenital malformations in infants of folic acid-deficient mothers (3.0%) than in a control group (1.6%) (162). Folate transfers and processes one-carbon unit coenzymes and synthetes thymidine nucleotides, which are essential for *de novo* DNA construction or repair, and are required for cell division and cell maintenance. In addition, folate is a key factor in “site-specific” methylation of cytosine bases in DNA, which regulates the expression of epigenetic genes (163). Folic acid has a preventive effect on reducing congenital heart disease, as has been reported in other studies (164). From a population-based Atlanta Birth Defects Case-Control Study showed mothers who reported periconceptional multivitamin use had a 43% lower risk of having infants with conotruncal defects (odds ratio [OR], 0.57; 95% confidence interval [CI], 0.33 to 1.00) than did mothers who reported no use (160). In a study of folic acid antagonists during pregnancy and the risk of birth defects, folic acid antagonists not only increased the risk of neural tube defects, but also increased the risk of cardiovascular defects. A multivitamin folic acid component may reduce the risk of these defects (165). In a systematic review of observational studies, the authors evaluated the effects of eight maternal micronutrients - vitamin D, vitamin B12, folate, vitamin A, zinc, copper, selenium, and ferritin - on congenital heart disease (166). Unfortunately, there is insufficient evidence to conclude with confidence whether maternal micronutrient deficiencies increase the risk of foetal CHD. Although further large-scale prospective studies are needed to confirm this contention, available data support that folic acid and multivitamins are essential for proper foetal heart development during early embryonic development and that supplementation of these substances during the peri-pregnancy or early pregnancy may reduce the risk of congenital heart disease (167). Other nutrients such as carbohydrate, protein and fat may not have direct evidence of their relationship with hereditary heart disease, but the deficiency of some nutrients may lead to impaired heart function, abnormal blood pressure and heart rhythm, which may affect the progression and symptoms of hereditary heart disease. Therefore, for patients with hereditary heart disease, it is still necessary to correct the nutrient deficiency by proper diet. This will improve symptoms and promote heart health.

## Conclusions

**Figure 4:**
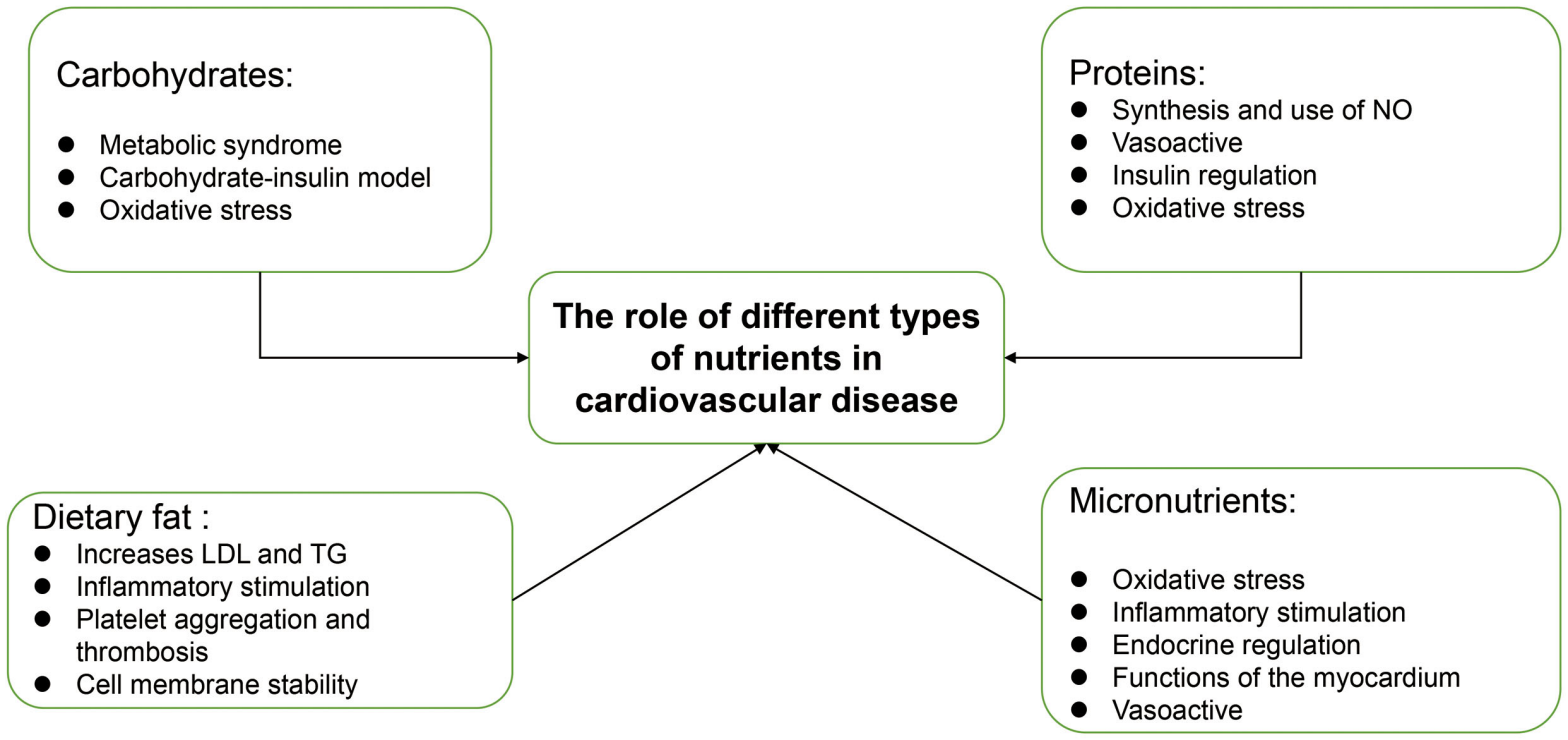
Impact of different nutrients on cardiovascular disease.

This review describes the types of macronutrients and micronutrients commonly found in the human body, their physicochemical properties and their metabolic characteristics. The pathogenesis of different nutrients in cardiovascular diseases is discussed and the effects of different nutrients on cardiovascular diseases are analysed (**Figure 4**). At the same time, we provide epidemiological evidences on the therapeutic and preventive roles of nutrients in different types of cardiovascular diseases, which is believed to be helpful in analysing and comparing the effects of different nutrients on different types of cardiovascular diseases. In addition to cardiovascular disease in general, an attempt has been made to analyse the role of nutrients in hereditary heart disease, and although there is only a small amount of evidence, we have been able to find a relationship between nutrient deficiencies and hereditary heart disease. Although we have analysed the effects of different nutrients on cardiovascular disease, the analysis has been limited to a single nutrient or food group. There is still a great deal of room for research on the effects of different food groups and nutrient ratios in the daily diet on cardiovascular health. In addition, although the metabolism of nutrients in the human body is very complex and is not described in this paper, it is interesting to see whether the interactions of different nutrients in human metabolism are involved in the development of cardiovascular diseases. In conclusion, nutrients have a very important impact on the occurrence, prevention and treatment of cardiovascular diseases, while the mechanisms of nutrients in cardiovascular diseases, as well as dietary patterns, nutrient combinations for cardiovascular health diets need to be further studied.

## Author contributions

ZT: Data curation, Formal analysis, Writing – original draft. JY: Methodology, Writing – review & editing. CF: Conceptualization, Supervision, Writing – review & editing.
